# Ferroptosis: CD8^+^T cells’ blade to destroy tumor cells or poison for self-destruction

**DOI:** 10.1038/s41420-025-02415-x

**Published:** 2025-04-01

**Authors:** Yuan Liang, Yixin Zhao, Zhaoyang Qi, Xinru Li, Yuguang Zhao

**Affiliations:** Cancer Center, the First Hospital of Jilin University, Changchun, Jilin, China

**Keywords:** Tumour immunology, Immune cell death, Targeted therapies

## Abstract

Ferroptosis represents an emerging, iron-dependent form of cell death driven by lipid peroxidation. In recent years, it has garnered significant attention in the realm of cancer immunotherapy, particularly in studies involving immune checkpoint inhibitors. This form of cell death not only enhances our comprehension of the tumor microenvironment but is also considered a promising therapeutic strategy to address tumor resistance, investigate immune activation mechanisms, and facilitate the development of cancer vaccines. The combination of immunotherapy with ferroptosis provides innovative targets and fresh perspectives for advancing cancer treatment. Nevertheless, tumor cells appear to possess a wider array of ferroptosis evasion strategies compared to CD8^+^T cells, which have been conclusively shown to be more vulnerable to ferroptosis. Furthermore, ferroptosis in the TME can create a favorable environment for tumor survival and invasion. Under this premise, both inducing tumor cell ferroptosis and inhibiting T cell ferroptosis will impact antitumor immunity to some extent, and even make the final result run counter to our therapeutic purpose. This paper systematically elucidates the dual-edged sword role of ferroptosis in the antitumor process of T cells, briefly outlining the complexity of ferroptosis within the TME. It explores potential side effects associated with ferroptosis-inducing therapies and critically considers the combined application of ferroptosis-based therapies with ICIs. Furthermore, it highlights the current challenges faced by this combined therapeutic approach and points out future directions for development.

## Facts

- The combination of inducing ferroptosis in tumor cells and immunotherapy holds a bright prospect for antitumor effects.
- Tumor cells exhibit higher ferroptosis resistance and possess more abundant means of ferroptosis escape compared to CD8^+^T cells.
- Derivatives of tumor cells undergoing ferroptosis can inhibit the antitumor effect of CD8^+^T cells.
- The characteristics of the tumor microenvironment not only lead to weakened antitumor immune effects but are also conducive to the immune escape of tumor cells.

## Questions

- Can tumor cells be specifically induced to undergo ferroptosis without affecting normal antitumor immune cells?
- How can we minimize the toxic and side effects caused by derivatives of ferroptotic cancer cells?
- How can ferroptosis be utilized to reverse the immunosuppressive microenvironment and protect the antitumor effects of immune cells?
- How can we design standardized and reasonable clinical translation experiments based on the atypia and heterogeneity of tumors, and explore reliable biomarkers to predict patients’ responses to ferroptosis-combined immunotherapy?

## Introduction

As a pivotal member of T cell subsets, CD8^+^T cells play a central role in the antitumor immune response. They can regulate the functions of other immune cells either by directly killing tumor cells or by secreting cytokines, thereby exerting antitumor effects [1]. However, tumor cells also possess cunning defense mechanisms, such as inhibiting the activation of antigen-presenting cells and regulating antigen expression, in order to evade the killing effects of CD8^+^T cells [2]. Therefore, one of the main focuses in the field of tumor immunotherapy is to strengthen the antitumor immunity of CD8^+^T cells and block the immune escape pathways of tumor cells. In recent years, with the emergence of immune checkpoint inhibitor (ICI) therapy, immune checkpoint molecules like Programmed cell death protein 1 (PD-1) on CD8^+^T cells have become important therapeutic targets. Blocking the binding of PD-1 to Programmed cell death 1 ligand 1 (PD-L1) with monoclonal antibodies can relieve the inhibitory effect of PD-1 on CD8^+^T cells and restore their antitumor activity, thereby enhancing the effectiveness of immunotherapy [3]. However, it is noteworthy that the therapy elicits a response in less than 30% of patients [4, 5], with the majority exhibiting treatment resistance and disease progression [6, 7].

**Fig. 1:**
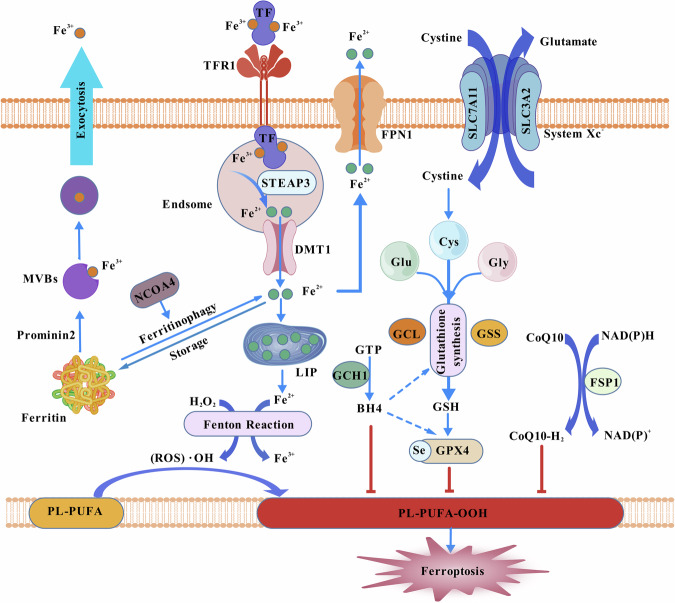
The main mechanism of ferroptosis. Intracellular iron accumulation: Intracellular iron accumulation is a critical determinant of ferroptosis. Endocytosis mediated by transferrin receptors, excessive release of ferritin leading to increased intracellular iron input, and reduced efflux mediated by ferroportin all contribute to intracellular iron overload, expansion of labile iron pool (LIP), and subsequent Fenton reactions resulting in lipid peroxidation of polyunsaturated fatty acids (PUFAs), ultimately culminating in ferroptosis. ROS production and increased levels of lipid peroxidation: Reactive oxygen species (ROS) induce an elevation in lipid peroxidation levels by reacting with PUFAs, resulting in the disruption of cellular membranes and triggering ferroptosis. Inhibition of the GSH-GPX-4 pathway: Glutathione (GSH) serves as a crucial intracellular antioxidant, collaborating with glutathione peroxidase 4 (GPX-4) to form a robust defense mechanism against lipid peroxidation. GPX-4, a kind of selenoprotein, utilizes GSH as a substrate to enzymatically reduce lipid hydroperoxides to their non-toxic counterparts, thereby preventing lipid peroxide accumulation and impeding ferroptosis. The tripeptide GSH, composed of glutamate, cysteine, and glycine, exerts antioxidant effects within cellular environments and serves as an essential cofactor for GPX-4. When GSH is depleted, or GPX-4 function is impaired, it leads to an exacerbation of lipid peroxidation reactions, thereby promoting the occurrence of ferroptosis. Inhibition of System Xc-: System Xc- is a transmembrane protein complex responsible for the cellular uptake of cystine and efflux of glutamate. Cystine serves as a precursor for GSH synthesis, highlighting the critical role of System Xc- in maintaining intracellular GSH levels. The decreased levels of GCH1-BH4: Tetrahydrobiopterin (BH4) is essential for maintaining GSH synthesis and the activity of GPX-4. Guanosine Triphosphate Cyclohydrolase 1 (GCH1) serves as the rate-limiting enzyme in BH4 biosynthesis. Reduced levels of GCH1-BH4 or dysfunction of this protein can increase cellular susceptibility to ferroptosis. Reduced levels of CoQ10: CoQ10 is a lipophilic antioxidant that plays a crucial role in maintaining cellular bioenergetics and redox balance, and exhibits some degree of inhibitory effect on ferroptosis. (Created with BioGDP.com).

Ferroptosis is a recently identified form of regulated cell death characterized by the accumulation of lipid peroxidation products and iron-dependent reactive oxygen species. Unlike classical forms of cell death, such as necrosis and apoptosis, ferroptosis is driven by an iron-catalyzed process [8]. Recent investigations have unequivocally demonstrated that the (i) intracellular iron accumulation [9], (ii) Reactive oxygen species (ROS) production and increased levels of lipid peroxidation [10], (iii) inhibition of the Glutathione- glutathione peroxidase 4 (GSH-GPX-4) pathway [11–14], (iv) inhibition of System Xc^-^ [8], (v) the decreased levels of Guanosine Triphosphate Cyclohydrolase 1-Tetrahydrobiopterin (GCH1-BH4) [15] and (vi) reduced levels of CoQ10 due to diverse factors [16], collectively contribute to the induction of ferroptosis (Fig. 1). With the continuous discovery of related mechanisms of ferroptosis, more and more scholars believe that regulation of ferroptosis combined with ICI treatment can enhance antitumor immune efficacy [17–19]. However, it is also important to consider that induction of ferroptosis may have implications for tumor immune evasion [20, 21]. This highlights the necessity for a comprehensive understanding of how ferroptosis impacts both tumor cells and immune cells in order to effectively harness its therapeutic potential.

## Ferroptosis of tumor cells

### Ferroptosis of tumor cells is the core of immunotherapy

Previous studies have demonstrated that CD8^+^T cells can induce cell death via the perforin-granzyme pathway and the Fas/FasL death receptor pathway, as well as indirectly kill tumor cells by releasing TNF-α [22–24]. Notably, Wang et al. identified a significant role of ferroptosis in CD8^+^T cell-mediated tumor cell killing, representing an additional mechanism for cytotoxicity. IFN-γ secreted by anti-PD-L1 immunotherapy-activated CD8^+^T cells markedly down-regulates SLC3A2 and SLC7A11 expression in tumor cells through the JAK1-STAT1 signaling pathway (SLC3A2 and SLC7A11 are the two subunits that constitute the cystine/glutamate antiporter, known as System Xc^−^), resulting in reduced cysteine uptake, compromised GPX-4 function, altered mitochondrial activity, heightened lipid peroxidation, and ultimately ferroptosis induction [17, 25]. Additionally reported is the direct induction of cancer cell ferroptosis by IFN-γ in an ACSL4-dependent manner when combined with arachidonic acid (AA). Specifically, IFN-γ activates the JAK/STAT1/interferon regulatory factor 1 (IRF1) signaling pathway to upregulate ACSL4 transcription in tumor cells by promoting IRF1 binding to ACSL4 promoter region IFN-stimulated response elements. In conjunction with AA presence, this process drives polyunsaturated fatty acids (PUFAs) into phospholipids (PLs) on the cell membrane to induce cancer cell lipid peroxidation and consequent ferroptosis [26–28].

Immunogenic cell death (ICD) is a distinct form of cellular demise characterized by the release of diverse damage-associated molecular patterns (DAMPs), which act as potent adjuvants to initiate an immune response [29, 30]. Currently, some scholars believe that ferroptosis is also a type of ICD. DAMPs derived from ferroptotic cancer cells, such as calreticulin (CRT), high-mobility group box 1 (HMGB1), and ATP, can engage with surface receptors on dendritic cells (DCs) including CD91, toll-like receptor 4 (TLR4), and purinergic P2RX7 receptor, thereby promoting DC activation and maturation to subsequently activate CD8^+^T cells [31–34]. The activated CD8^+^T cells, in turn, stimulate the adaptive immune system within the TME through IFN-γ production and mediation of antitumor immunity [35, 36]. What’s more, pro-inflammatory cytokines produced by DCs, such as IL-12, can directly influence CD8^+^T cells to enhance their proliferation and cytotoxicity [37]. Additionally, lipid peroxides generated by ferroptotic cancer cells may augment DC recognition and processing of tumor antigens while expediting the activation of CD8^+^T cells and cytotoxic T lymphocytes to impede tumor growth [38]. These mechanisms suggest that ferroptosis could serve as a pivotal “trigger point” for initiating the positive feedback loop involving CD8^+^T cell-mediated eradication of tumor cells, thus significantly enhancing antitumor activity.

A Phase 2 open-label cohort study demonstrated that the combination of pembrolizumab with chemotherapy exhibited significant advantages compared to chemotherapy alone [39]. However, the mechanisms underlying how platinum-based chemotherapy promotes antitumor immune responses remain incompletely understood. Existing literature suggests that the primary reason for chemotherapy’s enhancement of the immune response to ICIs lies in cisplatin’s ability to kill tumor cells by inducing ferroptosis. Furthermore, preclinical studies have confirmed that the synergistic antitumor effect of the combination therapy involving chemotherapy and ICIs is significantly inhibited after intervention with ferroptosis inhibitors, indirectly highlighting the crucial role of ferroptosis in clinical immunotherapy [40].

**Fig. 2:**
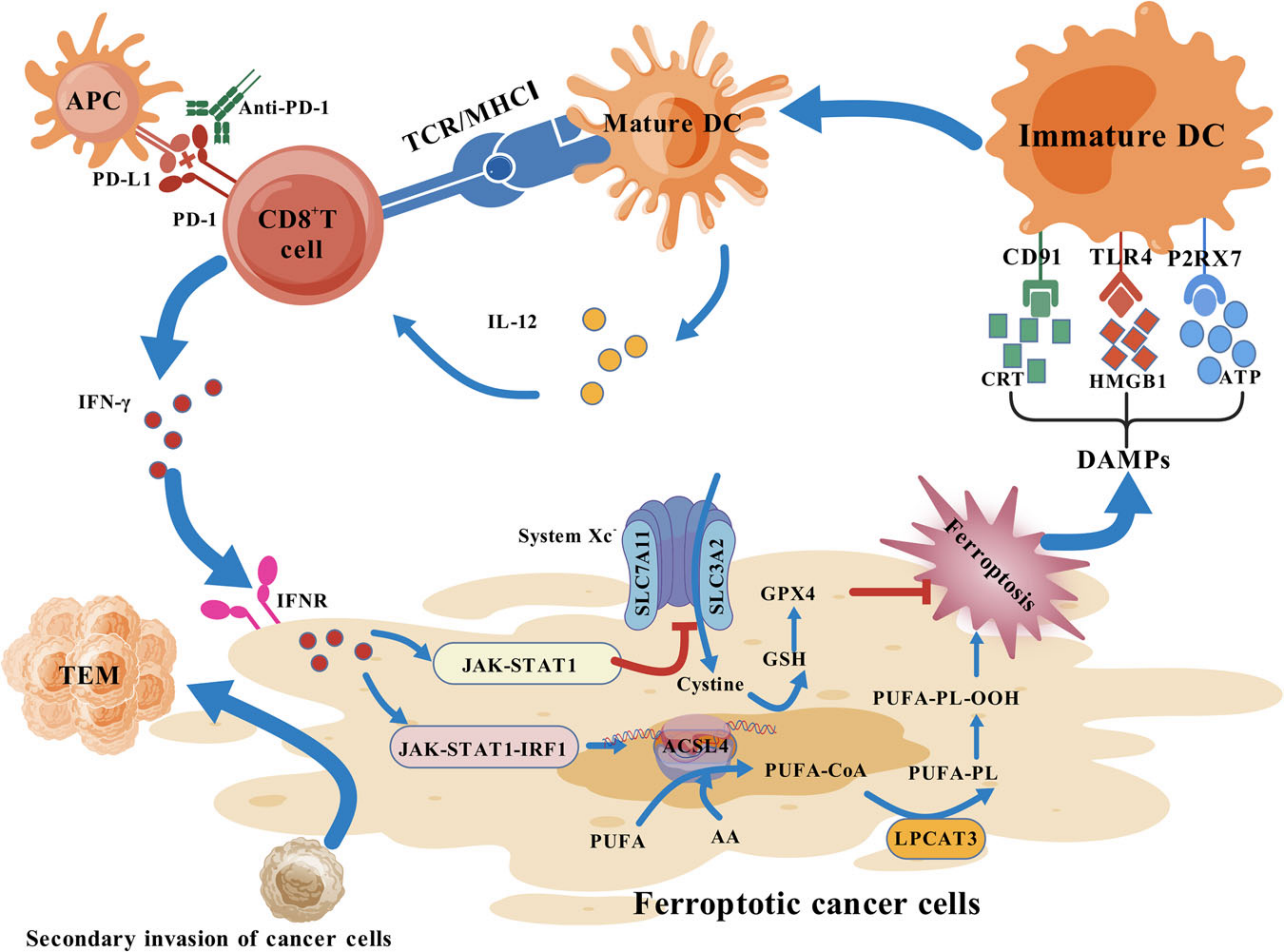
A positive feedback loop in which CD8^+^T cells kill tumor cells through ferroptosis. The IFN-γ released by CD8^+^T cells activated by anti-PD-1 therapy induces ferroptosis in tumor cells through the following two mechanisms: inhibiting System Xc- via the JAK-STAT1 pathway and promoting lipid peroxide production via the JAK-STAT1-IRF1 pathway. The DAMPs released during tumor cell ferroptosis can promote the maturation of DCs, thereby enhancing the antitumor effect of CD8^+^T cells, forming a positive feedback loop for tumor cell killing. Organisms that have undergone ferroptosis induction can generate a faster immune response through the amplification of TEM when facing secondary tumor cell invasion. (Created with BioGDP.com).

Additionally, the promotion of tumor cell ferroptosis appears to hold the potential for inducing immune memory. Li and Zhang’s study revealed that mice with tumors that had undergone ferroptosis-induced response were able to elicit a swifter immune reaction compared to the control group, characterized by an expansion of effector memory T cells (TEM) [41, 42]. Efimova et al. [43] demonstrated experimentally that early ferroptotic cancer cells are immunogenic and can both facilitate the maturation and activation of bone marrow-derived dendritic cells (BMDCs) in immunocompetent mice and accelerate the T-cell antitumor immune response (Fig. 2).

### The susceptibility of tumor cells to ferroptosis

There are significant differences in the susceptibility of different types of tumors to ferroptosis. Specifically, the vulnerability to ferroptosis can be elucidated through the following three characteristics: the metabolic features, gene expression profiles, and genetic mutation patterns of the tumors. Based on these characteristics, we can assess which ferroptosis-inducing therapies patients are likely to benefit from. For instance, compared with normal cells, tumor cells show an increased demand for iron to synthesize DNA and complete self-proliferation [44, 45]. However, the increase in intracellular iron leads to increased levels of ROS and lipid metabolites [46], which enhances the sensitivity of tumor cells to ferroptosis and may be a breakthrough point for the treatment of some iron-rich or ROS-rich tumors (such as HCC, PDAC, BC, and NSCLC) [47].

Viswanathan et al. found that mesenchymal cancer cells are more prone to ferroptosis than epithelial cancer cells and also show a high dependence on GPX-4 due to enhanced synthesis of PLs containing polyunsaturated fatty acid chains, resulting in a high sensitivity to GPX-4 inhibitors [48]. Metadherin plays an important role in inducing epithelial-mesenchymal transformation (EMT), proliferation, and invasion of tumor cells, promoting treatment resistance and mesenchymal hypercytosis while increasing the vulnerability of tumor cells to ferroptosis inducers, especially GPX-4 inhibitors. Specifically, this process is achieved by Metadherin downregulating GPX-4 expression and negatively regulating System Xc^-^ [49].

For specific tumor types, selectively eliminating cancer cells by targeting their vulnerability to ferroptosis can enhance therapeutic efficacy while minimizing adverse effects.

#### Lung cancer (LC)

Within the context of small cell lung cancer (SCLC), non-neuroendocrine SCLC cells demonstrate greater sensitivity to ferroptosis compared to neuroendocrine SCLC cells. This difference is partly due to the overexpression of ePL synthase and high intracellular levels of PUFA-ePL in non-neuroendocrine SCLC cells [50]. As for non-small cell lung cancer (NSCLC), mutations in the epidermal growth factor receptor render these cancer cells highly dependent on cysteine supply. When interfered with through SLC7A11 inhibition or cystine deprivation, these NSCLC cells exhibit significant sensitivity to ferroptosis [51]. Furthermore, cysteine desulfurase (NFS1), a crucial iron-sulfur cluster biosynthetic enzyme, is highly expressed in well-differentiated NSCLC. By maintaining the stability of iron-sulfur cofactors, it effectively protects cancer cells from the threat of ferroptosis under oxidative stress. Inhibition of NFS1 has been shown in vitro to induce ferroptosis and inhibit tumor growth, providing a promising and effective strategy for the treatment of NSCLC [52].

#### Hepatocellular carcinoma (HCC)

The loss of Rb protein function is a significant event in the progression of liver cancer. It triggers a marked increase in mitochondrial ROS concentration and exacerbates cellular oxidative stress responses, providing a potential biological basis for using sorafenib to treat HCC through the ferroptosis mechanism [53]. Additionally, the Sigma 1 Receptor (S1R), which is abundantly expressed in hepatocytes, has emerged as a notable target. Inhibiting the activity of this receptor has been shown to effectively promote ferroptosis in HCC cells [54]. Furthermore, targeting and downregulating a series of negative regulators of ferroptosis in HCC, such as Nuclear Factor Erythroid 2-related Factor 2 (NRF2), Metallothionein-1G (MT-1G), CDGSH Iron-Sulfur Domain 1 (CISD1), and P53, also offers a feasible approach to accelerate ferroptosis in HCC cells [55].

#### Clear cell renal cell carcinoma (ccRCC)

Due to the high expression of alkylglycerophosphate synthase, a key enzyme in the PUFA-ePL synthesis pathway, ccRCC cells exhibit elevated levels of PUFA-ePL, making them particularly sensitive to ferroptosis [56]. Furthermore, ccRCC cells rely heavily on the GSH/GPX-4 pathway to defend against lipid peroxidation and the threat of ferroptosis. Therefore, these cells show extreme sensitivity to the depletion of glutamine and cystine, which are substrates for GSH synthesis. Existing evidence suggests that inhibiting GSH synthesis within ccRCC cells can effectively induce ferroptosis and suppress tumor growth [57]. Under hypoxia, the hypoxia-inducible factor (HIF) is activated and involved in the regulation of angiogenesis, growth metabolism, and apoptosis [58]. Among them, HIF-2α activation upregulates the expression of Hypoxia-Inducible Lipid droplet-associated protein, drives the accumulation of PUFA and the occurrence of lipid peroxidation, and thus increases the susceptibility of tumor cells to ferroptosis. This process is particularly evident in VHL-defective ccRCC cells, potentially offering new perspectives for the treatment of ccRCC [57, 59].

#### Triple-negative breast cancer (TNBC)

Increased levels of PUFAs, expansion of the LIP, and a weakened GSH/GPX-4 defense system collectively determine the susceptibility of TNBC to ferroptosis [60]. Cysteine is one of the crucial amino acids in TNBC. When the activity of the system Xc^-^ is inhibited, the uptake of cysteine decreases, triggering ferroptosis [61]. Another study has shown that the expression of the MUC1-C transmembrane protein is significantly elevated in TNBC, functioning similarly to the xCT light chain of the system Xc^-^ and playing a vital role in maintaining GSH levels and redox balance. MUC1-C can bind to xCT and CD44 variants (CD44v) to form a complex, regulating GSH levels through its interaction with xCT. Blocking the activation of the MUC1-C/xCT signaling pathway can induce ferroptosis in TNBC cells, thereby killing the tumor cells or impairing their self-renewal capacity [62].

### Survival strategies of tumor cells in response to ferroptosis stress

Although tumor cells are susceptible to ferroptosis, they also have the ability to escape the threat of ferroptosis. Under prolonged treatment, certain tumor cells may resist ferroptosis-inducing therapies through adaptive mechanisms (such as enhancing antioxidant capacity and altering iron metabolism pathways), leading to treatment failure. A more comprehensive understanding of the mechanisms by which tumor cells adapt and develop drug resistance will pave the way for maximizing the effectiveness of ferroptosis-promoting therapies. Proteins such as Nicotinamide nucleotide adenylyltransferase 1, which are highly expressed in tumor cells and involved in the assembly process of mitochondrial Iron-Sulfur Cluster, as well as calcium-independent phospholipase A2β (iPLA2β), can reduce the sensitivity of tumor cells to ferroptosis by decreasing the LIP and hydrolyzing peroxidized lipids, respectively, leading to the evasion of ferroptosis in tumor cells [52, 63]. Conversely, inhibition of endogenous iPLA2β sensitizes tumor cells to p53-driven ferroptosis, but does not affect the viability or growth of normal cells [64]. The upregulation of the Xc^-^/GSH/GPX-4 axis is also an important mechanism for tumor cells to escape ferroptosis. Tumor cells can enhance the expression of SLC7A11 by deactivating tumor suppressor factors (such as P53, BAP1, and ARF) and activating KRAS expression [65–68], while high expression of GSH and GPX-4 is also confirmed in various tumor cells [69, 70]. NRF2 is the main regulator of antioxidant defense [71]. Generally speaking, Recombinant Kelch-like ECH-associated Protein 1 (KEAP1) can bind to NRF2 and promote the ubiquitination and rapid degradation of NRF2, thereby inhibiting NRF2 [72]. Under oxidative stress, NRF2 dissociates from KEAP1, resulting in SLC7A11 overexpression, which promotes cell resistance to ferroptosis [73]. In addition, antioxidant enzymes such as ferroptosis suppressor protein 1 (FSP1), which catalyzes the conversion of CoQ10 to ubiquinol, and GTP cyclohydrolase 1, which catalyzes the production of tetrahydrobiopterin, have been shown to be upregulated in some cancers, helping tumor cells escape ferroptosis and promote tumor development [15, 74]. Furthermore, environmental factors also contribute to the ferroptosis resistance of tumor cells. Oleic acid (a kind of MUFA) in lymph fluid can be used by cancer cells to protect themselves against ferroptosis in an ACSL3-dependent manner. Additionally, lymph with a high proportion of GSH/GSSG also reduces the oxidative stress level facing cancer cells [75, 76]. This explains why lymphatic metastasis is often preferred over hematogenous metastasis from the perspective of ferroptosis. While in the hypoxic tumor microenvironment, in contrast to the effects of HIF-2α, upregulation of HIF-1α in tumor cells enhances ferroptosis resistance by increasing cystine uptake, promoting SLC7A11 transcription, and lipid droplet formation [77, 78]. Interestingly, although CD8^+^T cell activation induced by ferroptosis can kill tumor cells by releasing IFN-γ [17], the presence of IFN-γ simultaneously leads to upregulation of PD-L1, which induces adaptive immune resistance and T cell dysfunction [79–81]. Dorand et al. [82] show that inhibiting cyclin-dependent kinase 5 is expected to break the awkward situation.

### Negative effects of ferroptotic cancer cells on T cells

As mentioned above, ferroptosis of tumor cells is the core of immunotherapy. Ferroptotic cancer cells can enhance the antitumor immunity of CD8^+^T cells directly or indirectly through multiple mechanisms. However, in addition to promoting the activation of CD8^+^T cells, the metabolites of ferroptotic cancer cells also have negative effects on the regulation of CD8^+^T cells to a certain extent. While HMGB1 acts as a kind of DAMPs to promote the activation and maturation of DCs, thereby enhancing the antitumor immunity of CD8^+^T cells, it can also function as a tumor-promoting factor, leading to immunosuppression. Tumor cell-derived HMGB1 has been shown to induce regulatory T cells (Tregs) to produce IL-10, which in turn suppresses CD8^+^T cell-dependent antitumor immunity [83]. Kusume et al. [84] have even demonstrated that HMGB1 may directly inhibit DCs, thus interfering with the host’s antitumor immunity.

Additionally, oxidized PLs produced by ferroptotic cancer cells can hinder the maturation and differentiation of DCs by downregulating the expression of the characteristic differentiation factor CD1a, exerting specific immunosuppressive effects on DCs. Bluml et al.’s study further confirmed that oxidized PLs can impair the function of CD8^+^T cells and suppress their proliferation. This process is achieved by oxidized PLs inhibiting histone H3 phosphorylation and limiting NF-κB recruitment to the IL-12 p40 promoter, resulting in reduced IL-12 production by DCs [84]. There is also evidence indicating that oxidized phosphatidylcholine can activate NRF2 and promote Th17 cell differentiation, thereby contributing to the inhibition of DCs maturation [85]. Dai et al. experimentally demonstrated that 8-OHG released by ferroptotic cancer cells promotes TAMs infiltration and M2 polarization by activating the stimulator of interferon gene (STING)-mediated DNA sensor pathway [86].

**Fig. 3:**
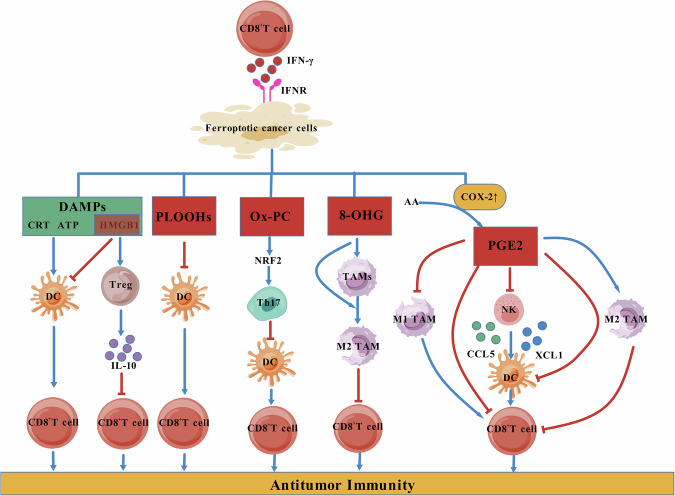
The influence of derivatives after tumor cell ferroptosis on CD8^+^T cells. The DAMPs released by ferroptotic cancer cells can enhance the antitumor immunity of CD8^+^T cells. However, HMGB1 among them, as well as other substances produced by ferroptotic cancer cells, such as PLOOHs, Ox-PC, 8-OHG, and PGE2 (which is synthesized through the upregulation of COX-2), can exhibit inhibitory effects on the antitumor immunity of CD8^+^T cells, either directly or indirectly. (Created with BioGDP.com).

It is noteworthy that AA metabolites also play a crucial role in immune regulation. Ferroptotic cancer cells can upregulate cyclooxygenase-2 (COX-2) expression and then elevate levels of its downstream product prostaglandin E2 (PGE2) [14], an important immune regulator capable of inhibiting chemokines CCL5 and XCL1 secreted by natural killer (NK) cells, consequently restricting cDC1 infiltration. Furthermore, PGE2 may directly impede cDC1 by reducing tumor-recruited chemokine receptor levels while affecting adaptive immune responses through cytotoxic T-cell function inhibition [87–90]. Konishi et al.’s experiments have observed that AA exerts inhibitory effects on CD8^+^T cells and DCs through another metabolic product from the COX pathway, thromboxane A2 [91, 92] (Fig. 3).

## Ferroptosis of immune cells

### CD8^+^T cell ferroptosis

The mechanisms of intracellular iron accumulation, increased levels of ROS and lipid peroxidation, and inhibition of the GSH-GPX-4 pathway are interconnected and collectively influence the fate of CD8^+^T cells. Intracellular iron accumulation can catalyze the Fenton reaction, promoting the generation of a large amount of ROS. Excessive ROS disrupts the original redox balance within the cell, exacerbating lipid peroxidation. Meanwhile, the GSH-GPX-4 pathway, serving as a crucial defense against lipid peroxidation, fails to effectively eliminate the produced lipid peroxides once it is inhibited [93]. This series of synergistic effects subjects CD8^+^T cells to higher oxidative stress, subsequently triggering ferroptosis, which leads to immune dysfunction and impairs their normal role in the antitumor process.

During T cell development, the proliferation of activated T lymphocytes relies on the provision of bioenergy by glucose and glutamine, while the excessive ROS generated during this process necessitates neutralization by endogenous antioxidants. To bolster their own antioxidant capacity, activated T lymphocytes accelerate their uptake of cysteine by upregulating the cysteine and cystine transporter to provide sufficient substrate for the synthesis of GSH. Although GSH deficiency may not impact early T cell activation, it can induce ferroptosis in proliferating T lymphocytes and compromise immune function [94, 95]. Yao et al. have suggested that augmenting GPX-4 levels through selenium supplementation under conditions of the presence of GSH could serve as an effective strategy to safeguard and enhance T cell immune function [96].

CD8^+^T cells can be categorized into subgroups such as Tc1, Tc2, Tc9, and Tc17 [1]. Notably, Xiao et al. observed that tumor-infiltrating Tc9 cells exhibited reduced levels of lipid peroxidation and exhibited enhanced antitumor effects compared to Tc1 cells. Studies have indicated that IL-9 secreted by Tc9 cells activates STAT3, which binds to the promoter of Carnitine Palmitoyltransferase 1 A (CPT1A), a key enzyme in fatty acid oxidation, inducing its transcription and upregulating CPT1A expression. The increased CPT1A activity promotes fatty acid oxidation and enhances mitochondrial function, thereby reducing their lipid peroxidation in TME to counteract ferroptosis [97]. Furthermore, IL-9 has been shown to enhance adaptive immune responses and antitumor effects through various mechanisms in certain tumors. Its potential application in adoptive cell therapy or targeting the IL-9/STAT3/fatty acid oxidation pathway may contribute significantly to existing immunotherapy strategies [98].

Enhanced lipid accumulation is a metabolic hallmark of the TME [99], necessitating upregulation of CD36 expression in CD8^+^T cells and other immune cells to facilitate the uptake and storage of fatty acids and cholesterol in response to environmental fluctuations. Cholesterol can induce PD-1 expression, impede CD8^+^T cell proliferation, and diminish levels of toxic cytokines (such as TNF-α, IFN-γ) by amplifying endoplasmic reticulum stress, thereby fostering immune evasion by tumor cells and functional impairment of CD8^+^T cells. Concurrently, CD36-mediated accumulation of AA and oxidized low-density lipoprotein (ox-LDL) can promote lipid peroxidation and ferroptosis in CD8^+^T cells [100, 101]. As a versatile transmembrane protein belonging to the scavenger receptor B class, CD36 plays a pivotal role in fatty acid transport, ox-LDL recognition, among others [102]. Moreover, CD36 can suppress TNF-α and IFN-γ production while attenuating the immune function of CD8^+^T cells [103]. Xu et al. demonstrated that ox-LDL uptake by CD8^+^T cells in a manner dependent on CD36 could induce p38 phosphorylation leading to self-consumption and loss of antitumor function. While overexpression of GPX-4 mitigated this decline through increased secretion of TNF-α and IFN-γ [100].

Recent researches have indicated a significantly lower expression of SLC7A11 in tumor-infiltrating CD8^+^T cells compared to tumor cells, suggesting a disadvantage for CD8^+^T cells in competing for cystine. This competition results in upregulated expression of CD36 in CD8^+^T cells, elevated oxidative stress levels, and accumulation of lipid peroxides, ultimately leading to ferroptosis. This process is accompanied by reduced secretion of cytokines, such as IFN-γ and TNF-α, as well as increased expression of PD-1 and TIM-3. Supplementation with cystine or forced expression of the glutamate-cysteine ligase catalytic subunit may represent potential therapeutic strategies to enhance T cell antitumor function [104].

Drijvers et al. observed that when co-cultured with cancer cells (B16, MC38), CD8^+^T cells exhibited higher sensitivity to RSL3-induced ferroptosis, resulting in a weaker tumor killing effect due to the elevated levels of ferroptosis in CD8^+^T cells compared to cancer cells. Although overexpression of GPX-4 in the cytosol, knockout of ACSL4, or the use of FSP1 can inhibit ferroptosis in CD8^+^T cells, the absence of the ACSL4 gene compromises the immune function of CD8^+^T cells. Therefore, maintaining a specific level of ACSL4 is crucial for regulating ferroptosis in CD8^+^T cells and preserving their functionality; it is not advisable to merely knock out ferroptosis-related genes [21].

### Ferroptosis of other immune cells

Although the regulation of ferroptosis has great potential in reversing the immunosuppressive microenvironment and enhancing the efficacy of T cells, its effects on other immune cells in the TME should not be ignored. Given that the interactions between Tregs, DCs, macrophages, myeloid-derived suppressor cells (MDSCs) and T cells are relatively close, the following paper mainly discussed the effects of ferroptosis on T cells within these four types of cells.

#### Treg ferroptosis

Tregs, a subset of CD4^+^T cells [105], can induce dysfunction in CD8^+^T cells and facilitate immune evasion by tumor cells through IL-2 (enhance the proliferation and cytotoxic activity of CD8^+^T cells) deprivation, upregulation of PD-1 and CTLA-4 expression as well as IL-10 and TGF-β secretion, among other mechanisms [106]. In the TME, Tregs often demonstrate greater resistance to ferroptosis compared to CD8^+^T cells due to their ability to secrete high levels of the antioxidant factor thioredoxin-1 to counteract ROS-induced ferroptosis [107]. Additionally, GPX-4 plays a crucial role in protecting activated Tregs from lipid peroxidation accumulation while maintaining their activation and function. GPX-4-deficient Tregs display heightened susceptibility to ferroptosis, undergoing lipid peroxidation accumulation and subsequent ferroptosis following TCR/CD28 co-stimulation. The literature confirms that GPX-4-deficient Tregs promote IL-1β production, inducing Th17 responses and activating DCs as well as CD8^+^T cells, thereby enhancing antitumor effects [108, 109]. However, it is important to note that ACSL4 inhibitors and System Xc^-^ inhibitors have minimal impact on inducing ferroptosis in Tregs [108]. For these reasons, many researchers consider GPX-4 inhibitors as a potential therapeutic strategy for promoting ferroptosis in Tregs while weakening immune suppression, thereby enhancing antitumor effects. It is worth noting that CD8^+^T cells exhibit higher sensitivity to GPX-4 inhibitors relative to tumor cells. Thus, the selective targeting of tumor-infiltrating Tregs ferroptosis remains an important research topic at present.

#### DC ferroptosis

As the primary antigen-presenting cells for T cells, DCs play a crucial role in not only presenting antigen peptides and MHC class I molecules to CD8^+^T cells, but also providing essential costimulatory signals for the activation of CD8^+^T cells, which is vital in initiating and regulating adaptive immune responses [110]. However, elevated lipid levels in the TME can promote ferroptosis of DCs and impair their function in tumor suppression [111]. Accumulation of ferroptosis regulators such as ROS and 4-HNE, a by-product of ferroptosis, can also induce endoplasmic reticulum stress and activate X-box binding protein 1, ultimately affecting antigen presentation by tumor-infiltrating DCs and subsequent activation of T cells [112, 113]. Additionally, Han et al. demonstrated that peroxisome proliferator-activated receptor-gamma (PPAR-γ), a nuclear receptor, can enhance RSL3-induced ferroptosis in DCs, leading to loss of their ability to express MHC class I molecules and stimulate IFN-γ secretion by CD8^+^T cells. Depletion of PPAR-γ may rescue DC maturation and function [114]. A recent study revealed that Sestrin2, a highly conserved stress-inducing protein, inhibits ferroptosis in DCs from septic mice by downregulating the ATF4-CHOP-CHAC1 signaling pathway, thereby improving the immune function of DCs [115]. While upregulation of Sesn2 may be an effective strategy to restore the antitumor activity of DCs in the TME. As DCs are of great significance to CD8^+^T cells, most of the rest of the content has been explained in detail in the previous discussion of CD8^+^T cells, so it will not be repeated here.

#### Macrophage ferroptosis

Tumor-associated macrophages (TAMs) can be classified into M1 and M2 subtypes based on their distinct functions and polarization. M1 TAMs secrete IL-12 and IL-18 to recruit and activate more CD8^+^T cells at the tumor site, promoting their proliferation, differentiation, and antitumor activity, indicative of immune activation [116]. Conversely, M2 TAMs release TGF-β and IL-10, restraining the maturation and activation of CD8^+^T cells and DCs, thus leading to immune suppression [117]. Typically, the TME harbors M2 TAMs, which contribute to an immunosuppressive milieu [118]. The process of ferroptosis, mediated by iron overload-induced ROS accumulation, has been shown to drive macrophage polarization towards the M1 phenotype through the ROS/acetyl-p53 pathway, thereby enhancing CD8^+^T cell infiltration in the TME [119, 120]. In addition, Zelenay et al. found that increased PGE2 due to ferroptosis of cancer cells not only stimulates the M2 phenotype, but also inhibits the M1 phenotype, which largely induces the occurrence of an immunosuppressive microenvironment [88]. It is important to note that M1 macrophages, due to their inducible nitric oxide synthase (iNOS) being expressed at higher levels, have higher levels of nitric oxide free radicals and are thus better able to inhibit lipid peroxidation. This explains why RSL3 can induce ferroptosis in M2 macrophages, but not in M1 macrophages [121]. The above conclusions undoubtedly provide a feasible therapeutic method to reverse the TAM phenotype in the TME and promote the antitumor immunity of T cells. In recent years, there has been increasing evidence that targeting macrophage ferroptosis therapy may be the next frontier in tumor immunotherapy [122].

#### MDSC ferroptosis

MDSCs, which are composed of (immature) monocytes and neutrophils, possess potent immunosuppressive capacity, inhibiting T cell activation and promoting the dysfunction in activated T cells [123]. Specifically, they can inhibit T cell proliferation and antitumor immune response through cysteine, arginine, and tryptophan deprivation, ROS release, and PD-L1 expression. In addition, they can also induce Tregs expansion by secreting IL-10 and TGF-β, thereby indirectly inhibiting T cell function [124]. With tumor progression, the metabolic reprogramming of MDSCs increases their uptake of exogenous fatty acid (FA) and further enhances the immunosuppressive activity of T cells [125]. Kim et al. demonstrated that polymorphonuclear MDSCs (PMN-MDSCs) in the TME are susceptible to ferroptosis due to hypoxia-mediated downregulation of GPX-4 and accumulation of AA resulting from upregulation of their own FA transporter protein 2. In general, ferroptosis of immunosuppressive cells can weaken the immunosuppression they cause. Surprisingly, PGE2 released by spontaneous ferroptosis of PMN-MDSCs inhibited CD8^+^T cells more strongly than it did in normal conditions [126]. In addition, the overexpression of N-Acylsphingosine Amidohydrolase 2 and aconitate decarboxylase 1 in MDSCs showed resistance to ferroptosis and drug resistance [123, 127]. Therefore, the application of ferroptosis inhibitors, rather than activators, is more conducive to reducing tumor immune escape and immunosuppression of T cells by MDSCs [123, 128].

## The impact of TME on tumor cells and related immune cells

Tumor cells, immune cells (including T cells, DCs, B cells, NK cells, macrophages), extracellular matrix, and soluble factors together constitute a complex interaction network of the TME [129]. Hypoxia, lactic acidosis, and lipid accumulation are unavoidable topics in the TME [99, 130].

Hypoxia often occurs due to increased metabolic activity of tumor cells and insufficient neovascularization density [131]. Hypoxia in the TME inhibits the activation of CD8^+^T cells, NK cells, and DCs, and promotes the activity of Tregs, the M2 phenotype polarization of TAMs, and the EMT process, which is mainly manifested as the weakening of the antitumor effect and the enhancement of immunosuppression [132].

The Warburg effect indicates that cancer cells prefer to obtain energy through glycolysis rather than mitochondrial oxidative phosphorylation even when oxygen is sufficient, and the unrestrained proliferative nature of tumor cells as well as the hypoxic environment undoubtedly further exacerbates the accumulation of lactate in the TME [133]. Lactate not only inhibits IFN-γ production by NK and NKT cells, but also limits CD8^+^T cell infiltration into solid tumors and can be used by Tregs to inhibit CTL function. It also plays an important role in promoting the polarization of TAMs to the M2 phenotype and inducing the activation of MDSCs by activating HIF-1α [134–139]. In liver cancer cells, high lactate levels can increase the level of MUFAs in the cell membrane and down-regulate the expression of ACSL4 through the AMPK pathway, thus effectively inhibiting ferroptosis in tumor cells [140].

Lipid accumulation in the TME is caused by the disorder of lipid metabolism in tumor cells and a large number of lipid metabolites released after ferroptosis [141]. The survival, proliferation, and invasion of tumor cells depend on the uptake and consumption of exogenous fatty acids, which reprogram lipid metabolism to meet the high affinity for lipids. The excess lipids can be stored in tumor cells in the form of lipid droplets, which can not only reduce the susceptibility to ferroptosis by reducing the PUFA content on the cell membrane, but also enhance the invasion ability of tumor cells, thereby affecting tumor progression [141, 142]. Interestingly, Dierge et al. found that dietary n-3 and n-6 PUFA could inhibit tumor cell growth in a dose-dependent manner under acidic conditions, which may be related to their promotion of lipid peroxidation and ferroptosis in tumor cells [143]. However, the effect of dietary PUFA on immune cells is still a significant research gap, and further research is needed to explore. The effects of the upregulation of CD36, caused by lipid accumulation, and the increase of AA-related metabolites (such as PGE2 and TXA2) on relevant immune cells have been described in detail in the previous section, and will not be repeated here.

**Fig. 4:**
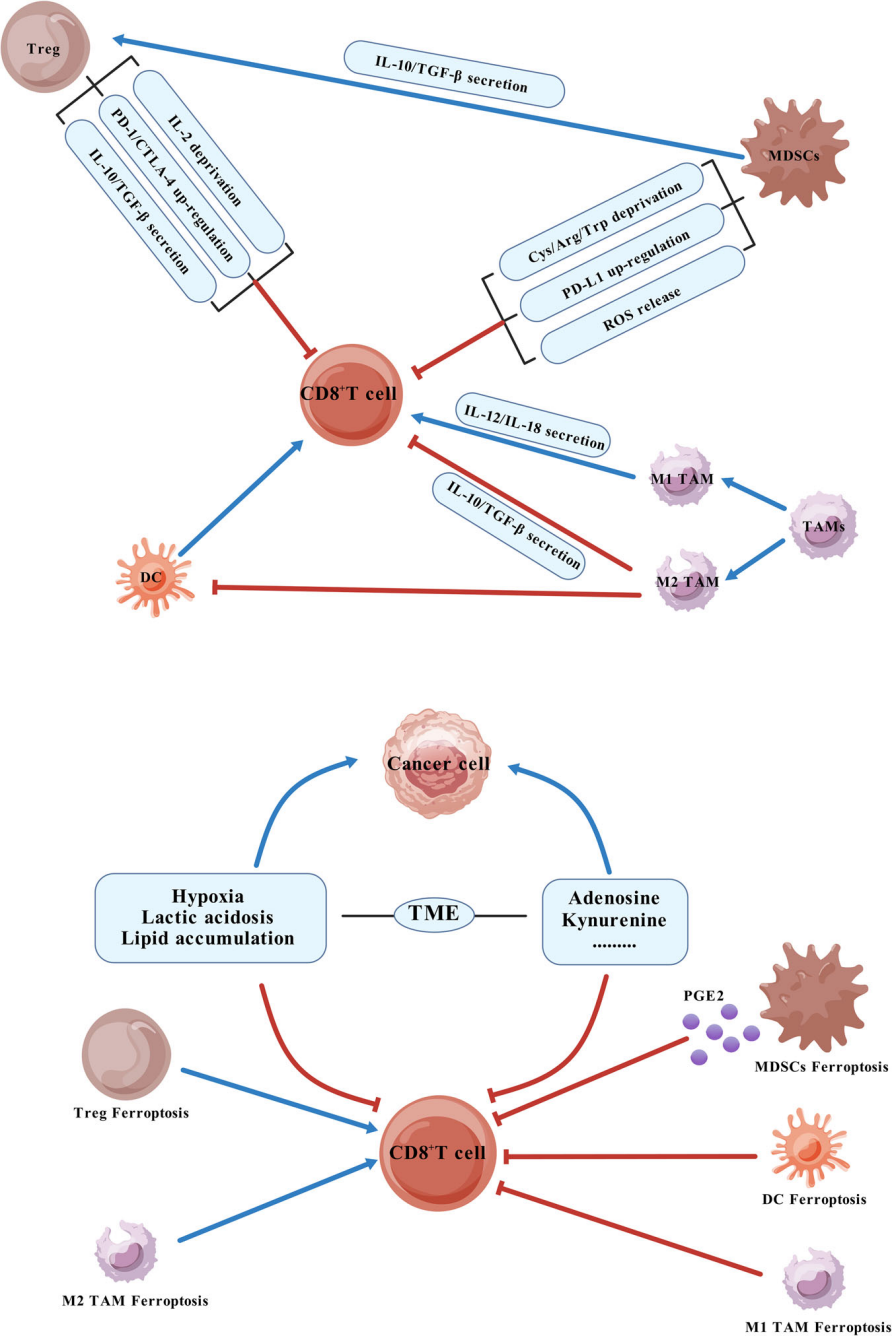
Relevant immune cells and TME’s impact on CD8^+^T cells. DCs and M1 TAMs exhibit a positive effect on the antitumor activity of CD8^+^T cells, whereas Tregs, M2 TAMs, and MDSCs exert opposite effects. Due to the release of PGE2 during MDSCs' ferroptosis, it demonstrates a more potent immunosuppressive effect than usual. The metabolic characteristics of the TME, including hypoxia, lactate acidosis, and lipid accumulation, as well as metabolites such as adenosine and kynurenine, collectively contribute to tumor promotion and enhanced immunosuppression. (Created with BioGDP.com).

In addition, some metabolites in TME, such as adenosine and kynurenine, have also been shown to negatively regulate the function of T cells and promote the immune escape of tumor cells [144, 145] (Fig. 4).

## The significance of ferroptosis in antitumor effects

### Ferroptosis plays a significant role in the immunotherapy of some cancers

As the most crucial component in immunotherapy, ICI therapy has been developed and received official approval from the US Food and Drug Administration (FDA) for clinical cancer treatment due to its durable efficacy, favorable safety profile, and reduced risk of recurrence [146–148]. This initiative has significantly altered the landscape of traditional treatments and ushered in a new era of antitumor therapy [149, 150]. It has brought long-term survival benefits to patients with certain types of cancer, particularly melanoma patients [151–155]. It cannot be overlooked that ferroptosis is essential for the antitumor efficacy of immunotherapy in melanoma. As discovered by Wang et al., the secretion of IFN-γ by CD8^+^T cells activated by immunotherapy, which promotes ferroptosis in tumor cells, is a crucial step for the effectiveness of immunotherapy [17]. Building on this, Qian et al. further confirmed this strong correlation between ferroptosis and immunity using bioinformatics. Their research showed that the upregulation of ferroptosis module genes is positively correlated with immune activation and is sensitive to anti-PD-1 treatment. Notably, this conclusion was also confirmed at a pan-cancer level [156]. Furthermore, studies have shown that in melanoma, upregulating TYRO3 to limit tumor ferroptosis promotes resistance to α-PD-1/PD-L1 immune checkpoint inhibition [157], while enhancing tumor ferroptosis by targeting the Wnt/β-catenin-MITF pathway can improve the efficacy of anti-PD-1 immunotherapy [158]. These two studies once again confirm the crucial role of ferroptosis in immunotherapy from both positive and negative perspectives. In various cancers, including colorectal cancer, the expression of DEPDC5 is closely correlated with the infiltration level of CD8^+^T cells within tumors, and its absence can weaken the antitumor immune response. Mechanistically, DEPDC5 can reduce the high levels of xanthine oxidase and lipid ROS produced by CD8^+^T cells, thereby shielding them from ferroptosis. This presents a novel strategy: enhancing antitumor immunity through the inhibition of ferroptosis [159]. The ferroptosis score (FPS) model developed by He et al., based on the ferroptosis database FerrDb, effectively evaluates the ferroptosis status in cancer patients [160, 161]. This model reveals a strong correlation between ferroptosis-related molecular features and the levels of immune cells, as well as treatment responses within the TME. It has been established as an important prognostic factor for assessing the efficacy of immunotherapy and serves as a reliable indicator for predicting the outcomes of cancer immunotherapy [162].

### Ferroptosis-based tumor vaccine

As previously mentioned, early ferroptotic cancer cells possess immunogenicity, which can promote the maturation and activation of BMDCs in immunocompetent mice, thereby enhancing T cell antitumor immunity [43]. However, Wiernicki et al. discovered that ferroptotic cancer cells not only fail to enhance the immune response of CD8^+^T cells, but may also inhibit antitumor immunity. When BMDCs were co-cultured with ferroptotic cancer cells, the maturity of BMDCs was negatively correlated with the early (1–2 h) ferroptosis of cancer cells. Although the markers of BMDC maturation increased during the middle (3–4 h) and late (5–8 h) stages, phagocytosis of ferroptotic cells significantly inhibited the expression of genes related to adaptive immune responses, which resulted in a weakened antigen presentation capacity. In a prophylactic vaccination model, although ferroptotic cancer cells released DAMPs and cytokines, they were unable to induce immunogenic protection against cancer cells at any stage of cell death [163]. It is apparent that the research findings of Wiernicki et al. contradict those mentioned in the previous text by Efimova et al. The main reason for this discrepancy is considered to be that the RSL3 used by Efimova et al. failed to induce complete ferroptosis in all the MCA205 cells subjected to the preventive vaccination, and the surviving tumor cells interfered with the experimental results to a certain extent [43, 163]. Of course, this inference is based on experimental processes and requires confirmation through further rigorous experiments. The development of ferroptosis-based tumor vaccines remains a viable option for future cancer immunotherapy.

### Immunotherapy combined with ferroptosis

Since the advent of ferroptosis in 2012, many scholars have regarded it as a potential strategy for antitumor therapy and set off a research upsurge in related fields. The combination of immunotherapy and ferroptosis provides a new way to enhance the antitumor effect of T cells. However, for now, there are still many problems to be solved in this treatment strategy. Whether ferroptosis serves as T cells' weapon to eliminate tumor cells or becomes their poison triggering self-destruction remains unclear. Through reviewing a large amount of literature, it can be seen that a large proportion of researchers have studied the above treatment methods locally and unilaterally. They focus more on discussing related content from the perspective of ferroptosis and tumor cells or from the perspective of ferroptosis and T cells, but often ignore the complex metabolic characteristics of the TME, the metabolic reprogramming of immune cells and tumor cells induced by it, and the crosstalk between other immune cells and T cells, as well as many other influencing factors. Therapeutically, the TME as a whole cannot be separated, so it is extremely important to balance the dual role of ferroptosis in tumor cells, antitumor immune cells, and immunosuppressive cells. Furthermore, numerous differentiating factors, such as the different types of tumors and various ferroptosis modulators, will serve as variables that can significantly impact the ultimate therapeutic effects. Even within the same type of tumor, individual differences among patients can influence their response to ferroptosis-inducing therapy. Specifically, factors such as heterogeneity in the tumor microenvironment, variations in metabolic characteristics, gene expression and mutation status, immune system activity, and prior treatments and drug responsiveness may all regulate the efficiency of ferroptosis induction in tumor cells and the therapeutic effects. While the complex immunosuppressive microenvironment, coupled with the intricate crosstalk between T cells and other immune cells, poses a significant obstacle to the clinical application of combined immunotherapy and ferroptosis-based treatment strategies. This also necessitates that, in future clinical applications of ferroptosis-inducing therapy, we conduct precise stratification based on the specific characteristics of patients to achieve more targeted treatment.

Ferroptosis-inducing therapy, as an emerging cancer treatment approach, has demonstrated significant potential in current antitumor research. However, its potential side effects and safety concerns cannot be overlooked. Existing literature indicates that inducing ferroptosis may lead to: (a) the death of immune cells, including CD8^+^T cells, DCs and NK cells; (b) stem cell death and bone marrow injury, with hematopoietic stem cells, due to their low protein synthesis levels, being particularly susceptible to ferroptosis inducers such as Erastin and RSL3. Additionally, the disruption of histone deubiquitinase MYSM1 during induction can trigger lipid peroxidation, further impairing their function; (c) liver and kidney toxicity, as these organs, being the most crucial metabolic organs in the human body, bear the risk of exposure to harmful substances during tumor treatment. Studies have shown that the conditional knockout of GPX-4 in the kidney or liver can lead to spontaneous ferroptosis damage; (d) cachexia, as demonstrated in an animal experiment where the induction of ferroptosis caused systemic responses, including increased lipid peroxidation and decreased NADPH levels, manifesting as early onset of cachexia and reduced survival rates; (e) secondary tumorigenesis, as some transgenic animal studies suggest that ferroptosis damage may play a potential role in initiating tumorigenesis, a process initially linked to the inflammatory response induced by ferroptosis [164]. To address these risks in treatment, Shi et al. proposed a precision medicine strategy for cancer treatment based on ferroptosis, which involves using multifunctional fluorescent probes to detect and monitor fluctuations in LPO, iron, ROS, and the cellular microenvironment during ferroptosis. This strategy envisions the application of ferroptosis probes for real-time treatment monitoring and intervention, image-guided drug delivery, and targeted therapy [165]. Additionally, monitoring liver and kidney function, assessing inflammatory markers, evaluating bone marrow and blood cell counts, and combining antioxidant drugs to prevent damage to vital tissues and organs are also effective clinical approaches.

Although the combination of ferroptosis and immunotherapy is still a controversial topic, based on the available research, the scientific community generally agrees that the promotion of antitumor activity exceeds the inhibition effect. Some of the research results have achieved remarkable results, which are worthy of our reference and continuous development. Cheu et al. found that immunotherapy, in synergy with the FSP1 inhibitor iFSP1, was able to significantly increase DC, macrophage, and T cell immune infiltration, and inhibit HCC progression. Unlike liver cancer cells, tumor-infiltrating immune cells overexpress GPX-4 but not FSP1, so iFSP1 selectively induces ferroptosis in cancer cells but not immune cells [166]. The findings of Cheu et al. suggest that identifying the relatively specific molecules expressed by tumor cells and focusing on their specific ferroptosis pathway theoretically provides the possibility to target ferroptosis of tumor cells and even specifically kill immunosuppressive cells by ferroptosis. In addition, the development of several small molecule agents, as well as nanoparticles, has shown surprising potential in reversing the immunosuppressive microenvironment and targeting ferroptosis in tumor cells [18, 167]. Moreover, adoptive cell therapy, through the adoptive reinfusion of antitumor immune cells modified with anti-ferroptosis properties and combined with ferroptosis inducer, can not only maintain the tumor killing effect of ferroptosis inducer, but also reduce the damage to antitumor immunity, thereby improving the efficacy of the combination of ferroptosis targeted therapy and immunotherapy.

## Summary and future perspectives

It is undeniable that the combination of ICI therapy and ferroptosis holds great promise in antitumor treatment. However, there are significant challenges that need to be addressed before its formal clinical application. Firstly, the regulation of ferroptosis involves a complex interplay of cellular metabolic pathways, such as iron metabolism, amino acid metabolism, and lipid metabolism, making it challenging to fully comprehend the regulatory mechanisms and effectively balance ferroptosis with ICI therapy. Additionally, the intricate crosstalk within the TME complicates the precise regulation of ferroptosis for specific cell types or subsets. Blindly manipulating ferroptosis without sufficient theoretical support is not advisable. Secondly, current studies often fail to account for the complexity of the TME and may yield inconclusive results due to inconsistent experimental methods. Therefore, continuous exploration of mature animal models, standardized experimental concepts, methods, and techniques is necessary. Lastly, thorough preclinical and clinical head-to-head studies are needed to carefully explore different types of ferroptosis regulators and determine their therapeutic windows for various cancer types over longer experimental periods, in order to assess efficacy, drug compatibility, drug resistance, and related side effects.
